# Meeting report: 2nd workshop of the United States culture collection network (May 19–21, 2014, State College, PA, USA)

**DOI:** 10.1186/1944-3277-9-27

**Published:** 2014-12-29

**Authors:** Kevin McCluskey, Scott Bates, Kyria Boundy-Mills, Arianna Broggiato, Anthony Cova, Philippe Desmeth, Chobi DebRoy, Deborah Fravel, George Garrity, María del Mar Jiménez Gasco, Lucy Joseph, Daniel Lindner, Michael W Lomas, Joe Morton, David Nobles, John Turner, Todd Ward, John Wertz, Aric Wiest, David Geiser

**Affiliations:** 1 University of Missouri- Kansas City, Fungal Genetics Stock Center, Kansas City, MO 64110, USA; 2 Department of Plant Pathology, University of Minnesota, 1991 Upper Buford Circle St. Paul, Minnesota, MN 55108, USA; 3 Phaff Yeast Culture Collection, University of California, Davis, CA 95616, USA; 4 Université Catholique de Louvain, Louvain-la-Neuve, B-1348 Louvain-la-Neuve, Belgium; 5 Addgene, 1 Kendall Sq. Ste, B7102 Cambridge, MA 02139, USA; 6 World Federation for Culture Collection, Belgian Sci. Policy Office, B-1050 Brussels, Belgium; 7 Penn State University, E.coli Reference Center, 115 Henning Building, University Park, PA, USA; 8 USDA Agricultural Research Service, GWCC-BLTSVL, 5601 Sunnyside Ave, Beltsville, MD 20705-5139, USA; 9 Department of Microbiology & Molecular Genetics, Michigan State University, East Lansing, MI 48824-4320, USA; 10 Department of Plant Pathology and Environmental Microbiology, Penn State University, University Park, PA 16802, USA; 11 Department of Viticulture and Enology, University of California, Davis, CA 95616, USA; 12 Center for Forest Mycology Research, USDA Forest Service, Madison, WI 53726, USA; 13 National Center for Marine Algae and Microbiota, Bigelow Laboratory for Ocean Sciences, East Boothbay, Maine 04544, USA; 14 Division of Plant & Soil Sciences, West Virginia University, 1090 Agricultural Sciences Bldg, Morgantown, WV 26506, USA; 15 University of Texas at Austin, The Culture Collection of Algae, Austin, TX 78712, USA; 16 USDA Animal and Plant Health Inspection Service, Biotechnology Regulatory Services, Riverdale, MD 20737, USA; 17 USDA Agricultural Research Service, USDA, ARS, MWA, NCAUR, BFPM, 1815 N University St, Peoria, IL 61604-3999, USA; 18 724 KBT, MCDB Department, E. coli Stock Center, Yale University, New Haven, CT 06520, USA; 19 Fungal Genetics Stock Center, Department of Plant Pathology, Kansas State University, Manhattan, KS 66506, USA

**Keywords:** Living microbe repository, Best practices, Regulatory compliance, Reference material collection

## Abstract

This report summarizes the activities and outcomes of the second workshop of the US Culture Collection Network, formally an activity of the US National Science Foundation sponsored Research Coordination Network for a Community of ex situ Microbial Germplasm Repositories. The workshop included presentations on topics as diverse as permitting for genetically engineered plant pest organisms to facilitating strain exchange via formal material transfer agreement systems and codes of conduct. Short talks introduced diverse collections held by government, university, and private entities. Participants visited living microbe collections as well as active research and production facilities.

## Introduction

Living microbe collections are an integral but often overlooked aspect of a mature research and development infrastructure and have been described as the foundations of the modern bioeconomy [1,2]. Living microbe collections, sometimes called culture collections or biological resource centers, hold and distribute authenticated living microbial cultures for a broad range of basic and applied research applications. Microbial genetics was foundational to early biological inquiry and spurred the transition from the natural history era to the biochemical genetic era. In the modern genomic era, sequenced genomes are more valuable when the living microbe strain is available for validation and experimentation, while metagenomic analyses depend upon the broad characterization of cultured reference strains [3]. Living microbe collections are recognized by the Convention on Biological Diversity as a key development tool [4] for sharing, preserving, and promoting utilization of microbial resources.

The US National Science Foundation has supported select living microbe collections for many years through the Division of Biological Infrastructure, and in 2012 funded a research coordination network (RCN) proposal for “A community of ex situ microbial germplasm repositories”. The exchange of best practices during meetings, workshops, and site visits was a key goal of the ambitious project. Additional goals included sharing information and protocols via a central website (http://www.usccn.org), development of shared informatics resources, and the implementation of best practices including off-site backup for active collections and the preservation of at risk or orphaned collections through off-site or inter-collection back-up.

This workshop was hosted by the Fusarium Research Center in the Department of Plant Pathology and Environmental Microbiology at the Penn State University, building upon prior meetings held at the Fungal Genetics Stock Center (5–7 September, 2012), the USDA NRRL collection (30–31 May 2013), and the National Center for Marine Algae and Microbiota at Bigelow Laboratory (9–10 October 2013). As with the previous meetings, this meeting accomplished several goals of the RCN grant including providing an opportunity for collection managers to meet with colleagues, funders, and regulators. Because more than half of the participants at this workshop had not participated in previous activities of the group, a thorough description of the origin of the group was presented including the historical role of the US Federation for Culture Collections, and recent efforts by the Ecological Society of America [5], and the American Phytopathological Society [6].

## Day 1

Recognizing that engagement of stakeholders is essential to the long term success of any infrastructure resource, the workshop brought together scientists working in a variety of capacities including regulation, funding, legal affairs, and collection development. For many participants, this was their first opportunity to interact with a group of collection managers outside their own institution.

Participants were welcomed by the Dean of the College of Agriculture as well as the Head of the Department of Plant Pathology and Environmental Microbiology. The Penn State University Plant Pathology department is among the oldest departments of its kind in the US and has housed working culture collections for many decades. Afternoon presentations included a description of the Fusarium Research Center and its development within the PSU Plant Pathology department.

Further presentations described the current regulations that govern movement of genetically engineered plant pests, as well as the development and organization of the US Plant Germplasm system. The USDA maintains hundreds of thousands of lines of cultivated plants and their wild relatives as well as germplasm for animal breeding and increasingly, microbial strains. Regulation of movement or release of acknowledged or potential microbial plant pests in the US is governed by the USDA Animal and Plant Health Inspection Service (APHIS). Within APHIS, Plant Protection and Quarantine (PPQ) is responsible for general permits specifying wild type strains while Biotechnology Regulatory Services (BRS) is responsible for permits for genetically engineered strains. Online “e-permitting” has simplified both processes and the USDA is committed to finding more effective ways to serve researchers whose laboratory, greenhouse, or small-scale field activities use regulated organisms, but who are not involved in larger scale commercial development or production activities.

Standards and codes of conduct have been used to provide authority and transparency to strain exchanges. Further presentations during the second afternoon session emphasized the development of formal codes of conduct for exchange of microbe strains. The current version of the code of conduct for exchange, called TRUST (for TRransparent User-friendly System of Transfer for Science & Technology), is built upon the frameworks stated by the MOSAIIC standard [1] and establishes proper practices for management of rights under the Nagoya Protocol and by extension, the Convention on Biological Diversity [7]. Because these issues are ultimately dependent upon accurate and properly curated reference material, further discussion included the use of standards in managing collection materials. Different standards were described including self imposed standards such as nomenclature [8] and also external standards for reference material, process optimization, and data management.

Finally, the third session showcased developments in management of yeast strain resources in the US and included descriptions of the University of California, Davis Phaff Yeast Culture Collection, which emphasizes biodiversity, and the UC Davis Wine Microbe Collection. Both collections maintain tremendous biodiversity, but have different research foci. For example, the Phaff Collection is comprised of over 7,000 isolates of over 800 species and many of these isolates are unique to this one collection. The Phaff collection is growing and developing strains from the collection for biofuel and other modern applications. The UC Davis Viticulture and Enology Wine Yeast and Bacteria Collection includes Saccharomyces and other yeast, fungi, and bacterial species associated with fermented beverages and is engaged in research and teaching relating to wine and other fermented beverage production.

## Day 2

Because many living microbe collection managers are more familiar with their own research community, the relationships established in the first day sessions were reinforced during discussions about how people exchange materials and the formal mechanisms adopted to facilitate these exchanges. It ended with introductions to collections managed by participants and visits to laboratories at Penn State University.

As the modern research enterprise becomes both more directly and implicitly collaborative, living microbe collections have an important role to play in assuring both that materials are accurately preserved and that research funds are not spent repeating prior experiments, but rather on building upon prior experiments. Because exchange of materials is essential to the progress of science, an analysis of current practices was conducted by Arianna Broggiato of the Centre for the Philosophy of Law, Universite Catholique de Louvain, Belgium. Results presented at this workshop included a comparison between responses from collections in the US and those in other countries, which showed that implementing best practices can facilitate exchanges and perhaps equally importantly, reinforces the awareness of exchange protocols for collection staff [9]. Such survey efforts are important as international exchanges of living microbe strains become governed by treaty law, such as the Convention on Biological Diversity or the Nagoya protocol [10]. Demonstrating the impact that collection networks can have on practices, this survey provides an important reference point for collections faced with increased responsibility as microbial materials are utilized for commercial and reference purposes.

To that end, material transfer agreements (MTAs) are a useful tool to help facilitate material exchange while preserving the rights of the providing and receiving parties. For example, since Addgene, a global, non-profit plasmid repository, distributes plasmids on behalf of its depositing institutions, all materials are sent out under a MTA to preserve the depositing institute’s rights in that material. Addgene has streamlined the MTA approval process by integrating the MTAs with their electronic request system. A significant advantage of this integration is that university technology transfer officers can approve sharing of molecular clones with a click of a button using the widely agreed upon and established terms of the NIH-authored Uniform Biological Material Transfer Agreement (UBMTA).The UBMTA creates a relationship between the depositing institution and the receiving institution. Addgene does not claim any materials rights in the materials deposited or any subsequent rights that may arise in inventions or modifications. This is fundamentally how the TRUST system envisions future exchanges; the TRUST system uses two connected documents: the MAA and MTA. The Material Accession Agreement (MAA) rules the deposit of material in a public collection and the MTA defines the conditions for further distribution. These documents are designed to complement one another and define rights as well as liabilities for exchange [11].

A goal of the microbial germplasm repositories participating in the RCN is to promote exchange and foster collaboration, and so the last formal session of the 2014 USCCN workshop at PSU included descriptions of several collections including the US Forest Service Culture Collection of the Center for Forest Mycology Research (CFMR) at the University of Wisconsin, Madison, and the University of Minnesota (UMN) Mycological Culture collection. The CFMR culture collection includes approximately 20,000 living cultures representing 1,600 species and continues to develop storage techniques for diverse fungi including tropical and temperate species. The UMN Mycological Culture Collection is associated with the Bell Museum of Natural History Herbarium that also houses approximately 100,000 dried fungal specimens in addition to over 1,000 living strains in the culture collection. Updates from the USDA NRRL collection and the Fungal Genetics Stock Center included the description of formalization of roles and funding streams at the USDA collection and the potential relocation of the FGSC. These two collections, the former emphasizing diversity of bacterial and fungal microbes, and the latter genetic research with fungi, hold over 150,000 isolates and distribute materials to all corners of the globe. Both have long histories and continue to make significant impact directly and in support of research in the areas of genetics, health, food, and fiber.

Additional presentations featured two collections located at Penn State that had not previously interacted with the collection community. The first, the *E. coli* reference center, holds over 70,000 isolates from human, animal, and environmental sources collected over the past 50 years. The lab provides diverse services, including classical serotyping as well as molecular genetic strain characterization.

The last presentation described a growing research collection of approximately 2,000 isolates of Verticilium, primarily *V. dahliae*, that was formed by merging collections from North America and Western Europe. This collection includes representatives of different vegetative compatibility groups and testers for characterizing newly isolated strains. Building upon these formal presentations, the group also visited the Fusarium Research Center, the PSU Mushroom Spawn Laboratory, the *E. coli* Reference center, and the PSU Creamery.

## Conclusion

This meeting marks the fourth time that US living microbe collection staff, curators, and directors were able to meet and visit each other’s laboratories since the research coordination network was initiated in 2012. Building upon the success of prior meetings, and in addition to formal presentations, time was budgeted both to visit local research and production facilities and to develop resources available via the central website of the network, (http://htpp://www.usccn.org). Of 22 formal participants, many were engaging in US culture collection network activities for the first time. Among these first-time participants, six were from universities in the US, three were from the US Department of Agriculture, one was from a non-profit collection, one was from a US Department of Energy laboratory, one was from a foreign university, and one from the World Federation for Culture Collections. This diverse group brings together knowledge of every domain impacting the activities of living microbe collections and emphasizes the impact and wide utilization of microbes in industry, medicine, agriculture, and the environment. Topics of emphasis at this meeting included permitting for genetically engineered plant pests, codes of conduct to satisfy international treaty obligations, material transfer agreements, and standards for data interoperability and portability. Presentations and visits described nine formal collections ranging from a laboratory based collection of approximately 2,000 strains of primary use by one research community to the USDA NRRL collection which includes approximately 95,000 strains in public, research, and patent collections of use in a wide variety of research and development activities including food safety and public health, plant and animal production, and biotechnological development (Table 1). Progress in the development of a community of living microbe collection workers has taken an important step toward the shared goal of a networked system of independent collections sharing infrastructure and resources by providing a forum for interaction and discussion.

**Table 1:** Online addresses of public collections described at the workshop

**Collection**	**Web URL**
Addgene Plasmid Repository	http://www.addgene.org/
Belgian Co-ordinated Collections of Micro-organisms	http://bccm.belspo.be/
Belll Museum of Natural History	http://www.bellmuseum.umn.edu/
E. coli Genetics Stock Center	http://cgsc.biology.yale.edu/
E. coli Reference Center	http://ecoli.cas.psu.edu
Fungal Genetics Stock Center	http://www.fgsc.net
Fusarium Research Center	http://plantpath.psu.edu/facilities/fusarium-research-center
International Culture Collection of (Vesicular) Arbuscular Mycorrhizal Fungi	http://invam.wvu.edu/
National Center for Marine Algae and Microbiota	https://ncma.bigelow.org/
Phaff Yeast Culture Collection	http://phaffcollection.ucdavis.edu/
PSU Mushroom Spawn Laboratory	http://plantpath.psu.edu/facilities/mushroom
The UC Davis Enology Culture Collection	http://wineserver.ucdavis.edu/industry/enology/culture/
USDA ARS Culture Collection	http://nrrl.ncaur.usda.gov/
USDA Forest Service Center for Forest Mycology Research	http://www.nrs.fs.fed.us/disturbance/invasive_species/herbarium_culture_collections/
UTEX Culture Collection of Algae	http://www.utex.org

## Competing interests

The authors declare that they have no competing interests.

## Authors’ contributions

KM presented at the meeting and drafted the manuscript, SB presented at the meeting and contributed significantly to the manuscript, KB-M presented at the meeting, AB presented at the meeting and contributed data and content to the manuscript, AC presented at the meeting and contributed to the manuscript, PD presented at the meeting, CDR presented at the meeting and shared data, DF presented at the meeting, GG presented at the meeting, MJG presented at the meeting and contributed to the manuscript, LJ presented at the meeting and contributed to writing the manuscript, DL presented at the meeting, MWL presented at the meeting and contributed to writing the manuscript, JM presented at the meeting and shared data, DN shared data for the manuscript, JT presented at the meeting and contributed to the writing of the manuscript, TW presented at the meeting, JW presented at the meeting and shared data for the manuscript, AW presented at the meeting, and DG presented at the meeting and contributed to the writing. All authors read and approved the final manuscript.
